# Indian Spring—Locution, Properties and Medicinal Virtues of the Water

**Published:** 1871-09

**Authors:** J. T. Banks


### INDIAN SPRING—LOCATION, PROPERTIES AND MEDICINAL VIRTUES OF THE WATER.

By J. T. Banks, M.D.

The Indian Spring is located in Butts County, Georgia, six miles southeast of Jackson, sixteen miles north of Forsyth, and twenty-two miles east of Griffin. The Spring is in a few yards of the junction of two little creeks that meet from opposite directions, and commingling their waters, unite and form one creek, that flows off at a right angle to the direction of both. The uninformed will often pass from one creek to the other, in their strolls along their shady banks, without noticing the change, until the current, wafting something on its surface, or the gentle rippling of the water, attracts the attention. It is probably the strongest sulphur spring in Georgia, and is composed of sulphate of magnesia, sulphate of potass, sulphate of lime, carbonate of magnesia, and three free gases—nitrogen, carbonic, and sulphuretted hydrogen; from the latter, it obtains its special unpleasant odor. The temperature of the water is sixty-three degrees. It is surrounded by a scenery *grand*, *inviting and entertaining*, in which Nature has furnished a psychical agent to the therapeutical virtues of the water. It is not my purpose to describe this scenery, if I could, and will refer to only one feature of it—a broken, rocky shoal, of about one hundred yards in length, and at least fifty feet fall—over which rushes one of the little creeks, which, by its continuous monotony, serves as a soporific to Indian Spring Visitors.

The quantity of water furnished by the Spring is small, but sufficient for a large number of persons. To see it, is to be reminded of an old aphorism, that "precious articles are put up in small bundles."

Owing to the chemical combinations and free gases in the water, it can not be transported and retain its special taste, much less its virtues; this I have carefully noticed. It soon loses the odor of sulpuretted hydrogen gas, and tastes flat and stale, and loses its medicinal virtues, however secure it may be corked and sealed.

It is a powerful eliminative agent, and begins its action very soon, either by the *skin*, *kidneys or bowels*. If the skin is hot and dry, the free use of it internally, aided by a warm bath of the water, will soon act beneficially. If there be a want of activity of the kidneys, a few small doses of morphine, combined with the water, soon aids in restoration of action. If there be habitual constipation, due to a sluggish action of bowels and intestinal mucous glands, the addition of a tea-spoonful of common salt, or of sulphate of magnesia, taken in the early morning draught, will soon correct it; or if due to a deficient hepato-pancreatic secretion, a small blue mass pill, taken two or three nights in succession, will aid the water in giving early relief.

It acts promptly as a soothing tonic to the organs of digestion, and a certain alterative in chronic inflammation of the stomach and liver. I have never failed to realize an improvement in my dyspeptic patients from the proper use of the water, unless they violated my hygienic instructions. Often the appetite improves faster than the digestive powers, and, unless controlled by prudence in eating, they may be overtaxed.

Convalescents from acute or chronic diseases of the abdominal or pelvic organs, or of the nervous system, are much benefited from its use. Much benefit is often obtained, also, from the judicious use of the electric baths, in conjunction with the water, in nervous affections, but they should never be used without the advice of a well-informed physician. One special destructive influence of this water should be well known: according to the observations of Dr. Whitehead, who has been there tor many years, and as he reports to me the same of Dr. Pitts, for many years before him, and other resident physicians, it has always injured tuberculous subjects, the diseased action being accelerated by the use of the water.

Its action is powerful and useful in the *diseases of females.* In amenorrhœa and the inflammatory form of dysmenorrhœa, it exercises a decidedly beneficial influence. I have often noticed its emmenagogue properties in my lady patients, sent there for its use; and ladies often tell me of its successful action in restoring menstruation after months' suppression, and after the long-continued use of medicine for that purpose. In the nausea and vomiting of pregnancy—especially extreme cases—it is the only remedy that I have ever obtained relief from, and in such cases, I never have had it to fail me. After trying alkalies, bismuth, cerium, carbonic acid, creasote, opiates, acids, tonics, etc., without any benefit, I have had every symptom relieved in a few hours by this water, *drank at the Spring;* and so well known is this special virtue in the neighhood, that often negro men may be seen with their jugs at night, for their wives under such circumstances. It is to this valuable property I would especially call the attention of my professional brothers. Some women obtain immediate relief, which continues during their use of the water, but have a return of all their trouble as soon as they leave the Spring; others obtain complete relief. I will not attempt to give its *modus operandi* at this time. One other benefit I have often observed with patients convalescing from chronic leucorrhœa—either vaginal or from disease of the os and cervical canal—

is, the prompt arrest of the discharge from its use as a vaginal douche three or four times per day.

In my opinion, there is no mineral spring in our country more deserving our professional attention than this, and I am truly glad to be able to inform the interested that the near approach by rail will soon be accomplished.

